# BH4-mimetics and -antagonists: an emerging class of Bcl-2 protein modulators for cancer therapy

**DOI:** 10.18632/oncotarget.26250

**Published:** 2018-10-19

**Authors:** Adrien Nougarède, Ruth Rimokh, Germain Gillet

**Affiliations:** Univ Lyon, Université Claude Bernard Lyon 1, INSERM 1052, CNRS 5286, Centre Léon Bérard, Centre de Recherche en Cancérologie de Lyon, Lyon, France

**Keywords:** Bcl-2 proteins, cancer, apoptosis, BH4 domain, therapy

**Figure 1:**
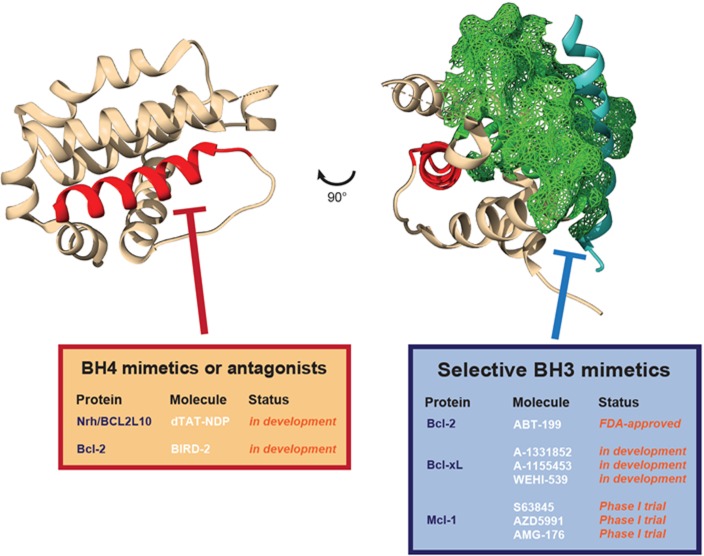
Differential targeting of distinct pro-survival Bcl-2 protein domains Representation of a typical Bcl-2 protein structure (here Nrh/Bcl2L10/Bcl-B, PDB: 4B4S). In red, the N-terminal BH4 helix. In blue, the BH3 alpha helix of Bim bound to Nrh. In green (mesh), the surface of the hydrophobic BH3 binding pocket of Nrh. The different selective molecules for the separate targeting of pro-survival Bcl-2 protein BH4 domains (BH4 mimetics or antagonists) or hydrophobic BH3 binding pocket (BH3 mimetics) are listed in the tables.

Dysregulation of cell death pathways is a hallmark of cancer progression. Hence pharmacological intervention to restore the apoptotic balance in tumors is considered to be a major objective for current cancer treatment strategies. At the molecular level, apoptosis is orchestrated by pro-apoptotic Bcl-2 family proteins and relies on the dual performance of Bax and Bak proteins, upon activation by BH3-only proteins Bim, Bid or Puma, leading to cell death via mitochondrial outer membrane permeabilization. As a safeguard mechanism, cancer cells rely on Bcl-2 pro-survival proteins (Bcl-2, Bcl-xL, Mcl-1, Bcl-W, Bcl-2A1/Bfl-1 and Nrh/Bcl2L10) to directly bind to the BH3 domain of Bax and Bak, as well as that of BH3-only proteins, via the hydrophobic groove formed by their BH3, BH2 and BH1 domains [5]. From this valuable knowledge gathered during the last three decades, the “BH3-mimetic” class of small-molecules was designed to mimic BH3 binding to the hydrophobic groove of pro-survival Bcl-2 proteins (see Figure 1 below). Currently, these molecules represent the main strategy to restore apoptosis either in combination therapy applications by only inhibiting pro-survival Bcl-2 proteins, or as single therapy agents able to displace Bcl-2-bound Bid, Bim or Puma to re-activate Bax and Bak.

However, growing evidence suggests that limiting the role of pro-survival Bcl-2 proteins to their sole capacity to inhibit Bax, Bak and BH3-only proteins might be incorrect. Indeed, Bcl-2 and Bcl-xL have also been shown to regulate calcium handling at the Endoplasmic Reticulum (ER), mostly by interacting with the Inositol 1,4,5-triphosphate Receptors (IP3Rs). Recently, our group demonstrated that another pro-survival Bcl-2 homolog from the Bcl-2 family, namely Nrh/Bcl2L10 or Bcl-B, could also interact with IP3Rs via its N-terminal BH4 domain. In contrast to Bcl-2 and Bcl-xL, Nrh is mainly found at the membrane of the ER where it was shown to negatively regulate apoptosis induced by thapsigargin or chemotherapeutic drugs by suppressing IP3Rs-mediated calcium signaling (Nougarede et al., 2018). Our study provided a much needed mechanistic insight into the newly uncovered role of Nrh in multiple myeloma [2]. Indeed, Hamouda and colleagues unveiled that the expression of Nrh alone was sufficient to drive tumorigenesis of B-cells in a mouse model, an unprecedented characteristic for a pro-survival Bcl-2 protein, the expression of which is generally considered to be insufficient to elicit a fully transformed phenotype. Further strengthening the relevance of Nrh as a therapeutic target in cancer, its expression was detected in more than 45% of breast cancer patients in a large cohort and was associated with a higher rate of metastatic relapse (Nougarede et al., 2018). Based on the action of Nrh at the ER, we then proposed to use a peptide comprising the sequence of the Nrh BH4 domain (dTAT-NDP), as a decoy to release full-length Nrh from IP3Rs and restore apoptosis in cancer cells. This strategy, involving for the first time a true Bcl-2 protein “BH4-mimetic” molecule, provided promising results by potentiating the action of chemotherapeutic drugs both *in vitro* and *in vivo* [4]. Interestingly, the use of the sequence of the BH4 domain as a targeted therapy discriminates Nrh from other Bcl-2 proteins, since it was sufficient, when used alone, to prevent full-length Nrh binding without blocking IP3Rs. It should also be noted that a peptide derived from the Bcl-2 binding site of IP3R was developed to restore apoptosis and IP3R signaling impaired by Bcl-2. This peptide, referred to as BIRD2, acting in fact as a “BH4-antagonist”, was used successfully to trigger apoptosis of human primary CLL cells [3]. Moreover, the selectivity of Bcl-2 family proteins towards specific IP3R isoforms, namely Bcl-2 for IP3R2 and Nrh for both IP3R1 and IP3R3, provides an additional level of specificity for therapies targeting the BH4 domains of the Bcl-2 family of cell death inhibitors [1].

One of the challenges facing current BH3-mimetic therapies is the *de novo* or acquired resistance to selective inhibitors, which is due to the high level of redundancy of the hydrophobic pocket of Bcl-2 family proteins. Using BH4-mimetics or -antagonists could be a means of alleviating resistance to therapy and sensitizing cancer cells to apoptosis. This concept was recently illustrated by using the BIRD-2 peptide to sensitize diffuse large B-cell lymphoma cell lines that were resistant to the FDA-approved Bcl-2 specific inhibitor ABT-199, or Venetoclax [6].

Nevertheless, several ongoing issues have to be addressed to facilitate the transfer of BH4 modulators into clinical applications, including (i) gaining a better understanding of their pharmacodynamic and pharmacokinetic properties, and (ii) investigating the possibility of using BIRD-2 and TAT-NDP peptides directly or replacing them by other kinds of small molecules.

Finally, it is worth noting that BH4-mimetics or -antagonists are not an attempt to replace BH3-mimetic therapies in the future, but rather offer a highly specific complementary tool to improve the success of current anti-cancer strategies.
